# Mucus and mucins in diseases of the intestinal and respiratory tracts

**DOI:** 10.1111/joim.12910

**Published:** 2019-04-22

**Authors:** G. C. Hansson

**Affiliations:** ^1^ Department of Medical Biochemistry University of Gothenburg Gothenburg Sweden

**Keywords:** COPD, cystic fibrosis, mucin, mucus, ulcerative colitis

## Abstract

This review describes the organization and importance of mucus in the intestine and lungs in relation to the diseases cystic fibrosis (CF), ulcerative colitis and chronic obstructive pulmonary disease (COPD). The inner surfaces of the body are protected by mucus built around polymeric glycoproteins called mucins. In the disease CF, the small intestinal mucus is in contrast the normal attached to the epithelium, explaining the intestinal problems at this disease. The inner of the two mucus layers of colon is normally impenetrable to bacteria, keeping the commensals away from and protecting the epithelium. This impenetrable property is dependent on the bacterial composition and the host diet, observations that can explain the increased incidence of inflammatory bowel diseases in the western world as bacteria reach the epithelial cells in active ulcerative colitis. The respiratory tract is normally cleared by thick mucus bundles that moved by the cilia sweep the epithelial surface. In CF, the bundles are nonmoving already at birth. Cholinergic stimulations stop the bundle movement explaining some of the beneficial effect of anticholinergic treatment in COPD. In this disease as well as in more developed CF, an attached mucus layer is formed. This mucus has features similar to the protective inner colon mucus and is by this able to separate bacteria from the epithelial surface. When formed in healthy individuals this mucus can be coughed up, but in chronically diseased lungs, bacteria colonizing the mucus will remain in the lungs and the resulting inflammation contribute to the destruction of the lungs.

## Abstract 2

Content List ‐ 15th Key symposium ‐ “Innate immunity”.

## Introduction

Nature has essentially two ways of protecting individuals from their immediate neighbourhood. One is to shield the live cells of the body by carrying a coating by dead cells or a hard shell. In humans, this is exemplified by the skin, mouth and oesophagus, that is covered by multiple layers of dead cells. Another way is to coat the live cells with mucus. This is the case for the intestinal and respiratory tracts. That mucus is important is obvious for any physician, but the mechanism behind its protective effects is less evident. Mucus has been most difficult to understand in the large intestine as it harbours such a high number of commensal bacteria. A major step forward was taken in 2008, when we could publish that the large intestine has an inner mucus layer that effectively separates the bacteria from the epithelial cells 1. Before this was understood, bacteria were believed to be in direct contact with the epithelial cells. Today, our model of an inner colon mucus layer impenetrable to bacteria is well accepted.

The importance and problems encountered by mucus and mucins are evident for the general public at times of upper respiratory tract colds. Examples of more specific mucus‐related diseases are given in Table 1.

**Table 1:** Examples of mucus‐related diseases

Organs	Disease	Symptoms
Mouth	Sjögren's syndrome	Dry mouth
Nose	Rhinitis, cold	Snot
Lungs	Bronchitis	Cough
Lung	COPD	Cough, infection, respiratory insufficiency
Lung	Cystic fibrosis	Cough, infection, respiratory insufficiency
Lung	Asthma	Obstruction, status asthmaticus
Stomach	Stomach ulcer	Pain, bleeding
Small intestine	Cystic fibrosis	Pain, obstruction, pancreatic insufficiency
Small intestine/Colon	Crohn's disease	Pain, diarrhoea
Colon	Ulcerative colitis	Bloody diarrhoea

COPD, chronic obstructive pulmonary disease.

## Principles of mucus

Mucus is easily observed ones infected, but normal mucus is totally transparent and invisible due to its high water content. However, physicians performing colonoscopy always observe light reflections from the lumenal ‘roof’, something that should not be possible if there was not an attached mucus layer that kept water bound to the surface. The function of mucus can be explained along two principles: (i) Cleaning the surface by mucus washing away debris and (ii) Protecting the surface by mucus coating. For cleaning, the mucus is important as it helps to bind and collect debris and bacteria that are then moved away by a liquid flow. The mucus is secreted by the epithelial cells out into the lumen and by this generates a directional flow away from the host. To act by protection, the mucus has to be attached to the epithelial cells to remain and act as a coat. The normal respiratory tract is cleaned by washing, whereas the colon is protected by a mucus coating. As we will see, most organs can switch between these two principles in response to challenges, something that is important for physiology, but also for understanding disease.

Mucus is formed by and secreted from specialized cells called goblet cells at surfaces or similar cell types in specialized glands. These cells provide most of the around 30 proteins that make up the core mucus proteome 2. Mucus is more than 95% water, making water and ions just as important for controlling mucus properties. In most organs, ions are delivered by other cells than the ones that produce the mucus. Controlling and adapting mucus properties are thus highly dependent on ion channels and their activities, allowing the system to respond quickly.

Organizing something with 95% water is not easy. It requires a skeleton of large, long molecules and ways to organize the water. These functions are largely accomplished by the gel‐forming mucins. These large molecules assemble into enormous polymers that build a skeleton around which mucus is organized. Mucins are highly glycosylated, typically >80%, and as carbohydrates efficiently bind water, they are able to maintain the high water content of mucus.

## The mucins

**Figure 1:**
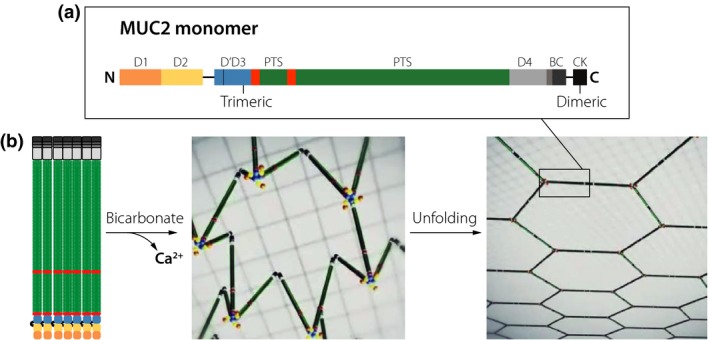
The MUC2 mucin structure, intracellular packing and unfolding upon secretion. (a) Domain organization of the 5130 amino acids of the MUC2 mucin, N, N‐terminal; C, C‐terminal; D. von Willebrand D domain; B, von Willebrand B domain; PTS, proline, threonine, serine sequence that after glycosylation become a mucin domain; CK, cysteine knot domain. (b) MUC2 mucin drawn to scale and packed in the secretory granule. After secretion, bicarbonate helps to remove calcium from MUC2 by increasing the pH and binding calcium allowing the mucin polymer to unfold into a flat net‐like structure that organizes the inner colon mucus layer.

There are four gel‐forming or polymeric mucins localized together on chromosome 11 in the human genome 3. These mucins have all evolved from the days when the first multicellular organisms developed, expanded dramatically in fishes and frogs and decreased in number in higher organisms when a more sophisticated immune system developed 4, 5. Goblet cells and their main product, the mucins, are early innate immune cells that are today highly integrated in our immune system 6. The more than 5000 amino acids in the human mucin monomers are organized with N‐ and C‐terminal parts that are involved in multimerization and a central part that carries most of the glycans (Fig. 1). This central part with all the *O*‐linked saccharides makes up more than 90% of the molecular mass and has a structure looking like a bottlebrush where the central extended protein core has the glycans extending out in all directions as the bottlebrush whiskers. The *O*‐glycans are attached to the serine and threonine amino acids of the central core and typically, these make up more than 50% of the amino acids in these so‐called PTS‐sequences. This fully glycosylated part forms a mucin domain that extent up to 0.5 μm in length and by all its glycans become like a stiff rod 7. As the glycans are attached to the protein core, they will bind and immobilize large amounts of water. Looking at a mucin from the outside, it is almost only glycans to see. The combination of large polymers and the water binding glycans together immobilize water to generate a diffusion barrier and give mucus their properties 8.

All four human mucins are covalently linked C‐terminal to C‐terminal via disulphide bonds, forming end‐to‐end dimers. This happens early during biosynthesis in the endoplasmic reticulum before glycans are attached. Later on, these dimers are sorted to specific vesicles in which these dimers form larger polymers, now by disulphide bonds between the N‐termini. In the MUC5B mucin, two are linked together 9; in the MUC2 mucin, three are linked together 7; and in the MUC5AC mucin, four are likely linked together. The MUC5B mucin thus forms linear polymers, the MUC2 flat net‐like polymers (Fig. 1) and the MUC5AC net‐like polymers after secretion.

Important for the release of these polymers is their well‐organized intracellular packing allowing them to be secreted and unfolded in an organized way (Fig. 1). The packing is caused by low pH and high Ca^2+^ concentration in these vesicles where a single bound Ca^2+^‐ion triggers a conformational change where the von Willebrand D1‐D2 domains fold back onto the D3 domain 7, 9, 10. This causes the MUC5B mucin to assemble into linear structures and MUC2 in ring‐like structures 7, 11. How the MUC5AC mucin is packed in the goblet cell granule is not known today.

The most important moment in the generation of normal physiological mucus is the secretion and the expansion from the intracellularly stored mucin to the fully expanded secreted one (Fig. 1b). This involves an at least 1000‐fold volume expansion. To accomplish this, the intracellular stored form has to be orderly packed allowing expansion without entanglement 7, 11. This also has to be relatively fast as noncovalent and covalent interactions and crosslinking between mucins will begin once secreted. Crucial for proper secretion is the quick removal of the Ca^2+^‐ion from the mucin, something that is accomplished by bicarbonate 12, 13. This ion will increase pH and help to precipitate out calcium. The bicarbonate is normally provided by the CFTR channel localized to goblet cell adjacent cells in organs affected by cystic fibrosis (CF). Insufficient amounts of bicarbonate cause poor and slow mucin unfolding that in the small intestine is directly linked to the disease 13.

## Small intestine protection and cystic fibrosis

**Figure 2:**
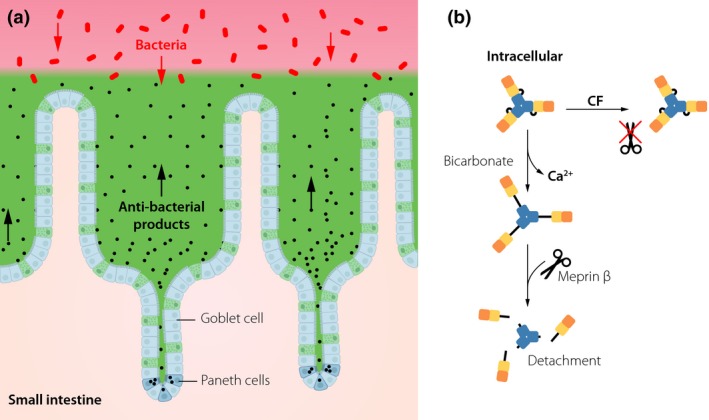
The mucus system of the small intestine. (a) All spaces between the small intestinal villi and over the villi tips are filled out by mucus generating a diffusion barrier that do not exclude bacteria by its pore size. Paneth cells in the crypt bottom secrete antibacterial peptides and proteins that together with the mucus keep the bacteria away from the epithelial cells. (b) The mucus of the small intestine is normally detached from its attachment by the meprin β protease cleaving off the N‐terminus of MUC2. Sufficient amounts of bicarbonate are required to unfold the MUC2 N‐termini and by this expose the meprin β cleavage site. In cystic fibrosis (CF), this unfolding is insufficient and renders the small intestine attached. Colours of the MUC2 N‐terminal domains as in Fig. 1.

The length of the villi of the small intestine varies and decreases in distal direction. Despite this, the volume between the villi and over their tips is filled with mucus along the whole small intestine (Fig. 2) 14. This mucus is rich in antibacterial peptides and proteins largely produced by the Paneth cells at the crypt bottom 15. Their antibacterial effect is explaining the limited contact between bacteria and the epithelium. The mucus is acting as a diffusion barrier that retains the antibacterial compounds at the intestinal surface with the highest concentration at the most vulnerable place, the crypt opening. The mucus will also limit the bacterial access by being a diffusion barrier, although the actual mucus pore size allows bacteria to enter.

The small intestinal MUC2 mucin is anchored to the goblet cells after secretion. Detachment from this anchor requires an active protease, meprin β 16. This enzyme cleaves the MUC2 protein in the von Willbrand D’ (VWD) domain and by this releases the VWD D1 and D2 assemblies, likely responsible for the mucin attachment to the goblet cells (Fig. 2b). Interestingly, in germ‐free animals the meprin β is retained in the enterocyte membrane and the mucus remains anchored to the epithelium. Upon bacterial colonization of germ‐free animals, the enzyme is secreted out into the mucus, but the mucus still remains attached for 4–5 week probably because the meprin β proenzyme is not activated 17. The importance of having a nonattached mucus in the small intestine is illustrated by the shift in intestinal microbiota. The attached mucus favours Bacteroidetes instead of Firmicutes bacteria, something that is reversed to the original composition once the mucus becomes detached. These observations suggest that the mucus attachment/detachment of the small intestine is well controlled by the host and highly important for maintaining the normal bacterial composition.

The inherited disease CF is caused by a nonfunctional CFTR channel. The disease severity largely comes from the lungs, but all organs producing mucus are more or less affected 18. Problems with the small intestine are affecting some CF individuals as they are born with meconium ileus or as adults develop the distal ileal obstruction syndrome 19. In both cases, mucus is accumulating in the distal ileum and typically, these patients require oral laxatives or in severe cases surgery. Although mice with a nonfunctional CFTR channel do not have the lung disease, they have a to humans similar small intestinal disease 20. If the mice are not on oral laxatives, they die from small intestinal mucus obstruction and local infection early in life. The small intestinal mucus is abnormal as it is attached to the goblet cells, similarly to germ‐free animals 13. However, this is not due to lack of meprin β as addition of recombinant enzyme does not cure these animals 16. However, adding bicarbonate does! This strongly suggests that the MUC2 mucin is not sufficiently unfolded in CF required for meprin β to reach its cleavage site(s) (Fig. 2b). This conclusion is also supported by the observation that the ileal mucus is attached in experiments where bicarbonate has been removed. Together these observations suggest that CFTR can modulate mucus attachment by affecting mucin unfolding and by this regulate mucus properties and provide the link between CFTR‐dysfunction and mucus pathophysiology in CF. It further suggests that CFTR regulation might be closely coupled to mucus property regulation.

## Colon mucus protection and ulcerative colitis

**Figure 3:**
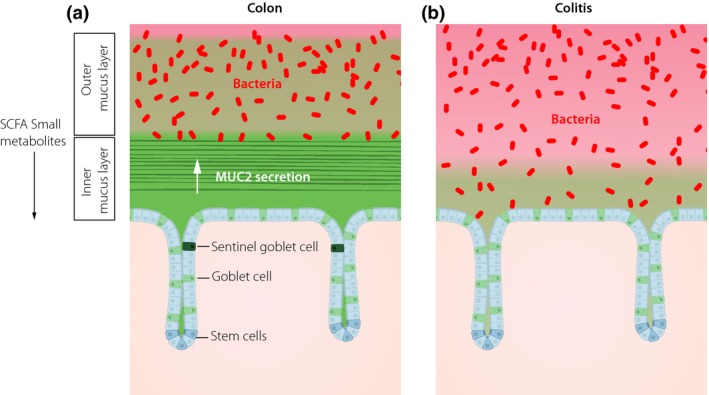
The mucus of the colon is two layered with an inner attached stratified mucus layer and a nonattached outer mucus layer. (a) The inner layer has pore sizes not allowing bacteria to enter, whereas the outer layer is expanded and generating the habitat for the commensal bacteria. Every crypt opening is guarded by a Sentinel goblet cell that senses when increased bacteria come in close contact and after activation coordinate compound exocytosis of a ring of goblet cells to generate a mucus plume moving bacteria away. The bacteria in the outer mucus layer generate short fatty acids (SCFA) and other metabolites that affect the epithelium. (b) In active ulcerative colitis, bacteria are reaching the epithelium due to failure of the inner mucus layer and loss of Sentinel goblet cells.

The large intestine is inhabited by an enormous number of commensal bacteria that belong to 1000 different species and by this carries more than a million genes as compared to the just above 20 000 human ones 21, 22. This generates a specific challenge to the host, especially as the bacteria divide and evolve fast. The outer colon mucus layer is the habitat for these bacteria where they meet the host‐generated mucin glycans at the outer surface of the inner mucus layer (Fig. 3a). The outer mucus layer is released from its anchor to the epithelium at this site and the coincident expansion of the net‐like polymer pore sizes allow bacteria to enter the mucus and by this get access to the mucin glycans. The commensal bacteria carry numerous glycosidases that remove monosaccharides from the mucin glycans one‐by‐one. However, few bacteria carry all enzymes requiring the bacteria to collaborate for the removal of all sugars and to reach the mucin protein core. There is one bacterium that might have all enzymes to accomplish a full mucin degradation; *Akkermansia muciniphilia* 23. The released monosaccharides are further metabolized in the bacteria and part of the obtained energy transferred back to the host in the form of the short fatty acids acetate, propionate and butyrate 24.

The inner mucus layer is the first line of colon defense against bacteria 1, 6. The inner mucus layer is built around the net‐like sheets formed by the MUC2 mucin stacked on each other to generate its laminated organization (Figs 1b and 3a) 7. The inner mucus layer is renewed from the epithelial surface goblet cells after the packed MUC2 has unfolded into sheets that interact with the already present inner mucus layer. This layer is anchored to the goblet cells as it cannot be flushed away. It acts as a physical filter where the pore sizes are smaller than 1 μm meaning that bacteria cannot penetrate into the inner mucus layer. The inner mucus layer is kept essentially free from bacteria and the layer generates a shield and protection for the underlying epithelial cells. The inner mucus layer in the distal colon is the thickest and is in live tissue measured to 50–70 μm in mice and 250–300 μm in humans 1, 25. The inner mucus layer is renewed in 1–2 h in the mice 26.

The second line of colon defense against bacteria is the Sentinel goblet cells where there is one cell guarding each crypt opening (Fig. 3a) 27. TLR2, 4 and 5 ligands are endocytosed by these cells, activate MyD88, Nox/Duox and finally activate the NLRP6 inflammasome and Caspase 1/11 causing Ca^2+^‐ion increase that is further transferred to and activating adjacent goblet cells. The Sentinel goblet cell and the surrounding goblet cells are undergoing compound exocytosis and the formed mucus plume will trap and move bacteria away from the crypt opening. Finally, the Sentinel cell is shooting itself out into the lumen. The sensitivity level for lipopolysaccharide is set so that the low levels found in and under the normal intact inner mucus layer do not trigger the Sentinel cell, but well the concentrations found in the lumen. When there are breaches in the inner mucus layer and bacteria come closer to the crypt opening, the Sentinel cell is activated. Continued activation depletes the Sentinel cells as renewal is slow, something that leaves the crypt opening without this defense. A third line of defense is likely organized by goblet cells in the crypt.

Ulcerative colitis is one of the two inflammatory bowel diseases. This disease has less of genetic influence than Crohn's disease and is thus more dependent on the environment and intestinal bacteria 28. This is further supported by the low frequency of ulcerative colitis in India and its rapid increase upon individuals moving to the United States 29. The restriction of ulcerative colitis to colon further suggests that its special mucus protective system could be of primary importance for the initial pathogenesis of this disease. This is supported by animal studies where a defective inner mucus layer that allowed bacteria to reach the epithelium was observed in all studied spontaneous mouse colitis models (Fig. 3b) 25. All humans with active ulcerative colitis also had a highly penetrable mucus where bacteria could reach the epithelial cells 25. All control humans with to ulcerative colitis unrelated diseases had bacteria well separated from the cells. Ulcerative colitis is a disease that comes and goes, and when patients in remission were studied, some of them had a to bacteria penetrable mucus (Fig. 3b). If bacteria reach the epithelial cells, they will more easily reach the lamina propria and its immune cells. The lamina propria harbours a population of self‐maintaining macrophages that can take care of a limited number of bacteria without triggering an immune response 30, 31. When also this defense line is overwhelmed by the bacterial load, there is activation of the adaptive immune system that triggers a typical overt immune response. Such responses will increase the levels of inflammatory stimulatory molecules and further trigger the secretion of mucus and contribute to the exhaustion of the mucus protection.

The importance of a protective inner mucus layer is emphasized by the understanding that its properties are highly dependent on the microbiota. Germ‐free mice have inner and outer mucus layers as in mice with normal bacteria, but the inner mucus layer is fully penetrable to beads the size of bacteria 17. Colonization of such animals renders the inner layer impenetrable after 6–7 weeks. However, this is dependent on the bacterial composition as with some bacterial communities this becomes only partly impenetrable directly mimicking the situation in the animals from where the bacteria were taken 32. However, it is not only the bacterial composition that determines the inner mucus layer properties. When animals were given a ‘western type’ diet with high fat, high sugar and low levels of by the host undegradable polysaccharides (fibres), the inner mucus layer becomes more penetrable to bacteria within three days 33. This happened without major changes in bacterial composition, suggesting that the bacterial metabolism was modified. Furthermore, adding a fructose polysaccharide called inulin partly rescued the mucus penetrability phenotype. Similar observations were made after measuring inner mucus layer thickness after fixation where it is known that the level of mucus shrinkage is related to mucus properties 34, 35. As an impenetrable inner mucus layer is dependent on bacteria that are present at a distance from the epithelium, there must exist bacterial metabolites that directly or indirectly stimulate the host epithelium to make an impenetrable mucus (Fig. 3a). To be able to diffuse through this mucus and against the mucus renewal gradient, these molecules are likely small. Recent studies of bacterial genomes have revealed that most bacteria contain gene clusters able to generate such molecules, not only short fatty acids but also more complex molecules often derived from amino acids 36. Such metabolites have been shown to have distant systemic effects on, for example, insulin‐producing cells, liver and maybe even the brain. It is thus likely that small bacterial metabolites also have local effects on the colonic epithelium and goblet cells 37. That the intestinal bacteria and their metabolism affect the protective capacity of the inner mucus layer may provide a first insight into why inflammatory bowel diseases are increasing in the western world.

The inner mucus layer is an essential system for protecting the colon epithelium and minimizing the bacterial contact. This system is highly dynamic, but has limitations in its capacity and speed by which it can replenish and generate a fully functional inner mucus layer. This makes the system prone to become exhausted and to fail.

## Normal cleaning of the respiratory tract by mucus bundles is stopped by acetylcholine and in cystic fibrosis

**Figure 4:**
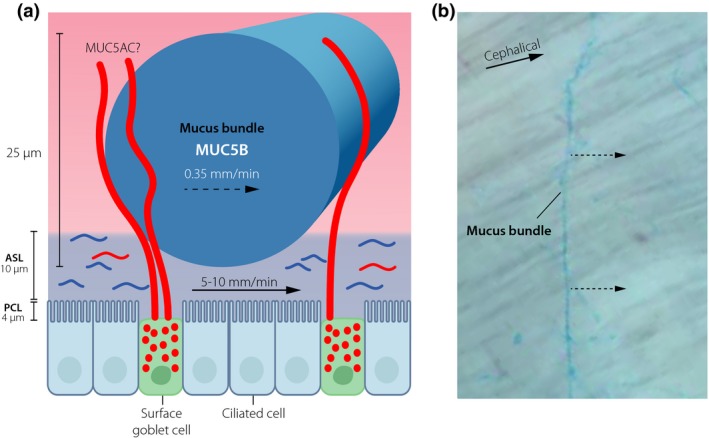
The mucus protective system of normal upper respiratory tract of higher animals. (a) Submucosal glands generate mucus bundles by >1000 MUC5B mucin linear polymers. The bundles are moved by the cilia and the air surface liquid (ASL). The mucus bundles are coated by the MUC5AC mucin from the surface goblet cells. (b) Mucus bundles stained with Alcian blue are moved cephalically over the tracheobronchial surface.

In contrast to the intestine, the lungs are kept essentially free from bacteria despite the high number of particles and bacteria that we inhale. These cannot be allowed to remain in the lungs and thus need to be cleared, something that is obtained by the escalator generated by the constantly beating cilia that is moving the air surface liquid (ASL) and mucus towards the larynx (Fig. 4). Although the cilia are similar in different species, the organization of the mucus varies as larger animals and humans have numerous submucosal glands, almost absent in mice 38. The submucosal glands produce long, thick mucus bundles that are then sweeping the tracheobronchial surfaces and efficiently clean the respiratory tract 39, 40. The mechanism for normal clearing of the lungs in higher organisms has been partly misunderstood as most conclusions have been made from chronically diseased lungs, cell cultures or rodents that have a different type of mucus clearance 41. Recent studies and obtained understanding are largely based on knowledge gained from studies on newborn piglets. This is important as these animals have not yet been heavily exposed to air pollution and have not generated the mucus coating found in diseased lung 39, 40, 42, 43.

The most distal cells in the submucosal gland secrete a chloride and bicarbonate‐rich fluid that flow through the gland canals 38. On its way, it passes by the cells producing the MUC5B polymeric mucin. This mucin is packed in the secretory granule of the cells with their N‐termini in central flower‐like structures due to the low pH and Ca^2+^‐ion concentration in these vesicles 9, 11. Once this granule is opened into the bicarbonate‐rich liquid, the Ca^2+^‐ions are released from the binding to the N‐termini and the packed MUC5B is pulled out into long linear molecules. The MUC5B mucin is held together in their N‐N and C‐C termini two‐by‐two and by this forms long covalent polymers 9, 11, 40. The liquid flow is fast and by this pulls out the MUC5B polymers that is moving slower through the gland ducts. The MUC5B polymers interact laterally and by this form thick bundles that at the gland opening reaches 25 μm in diameter and will contain 1000–5000 MUC5B polymers. These bundles have diameters that are much larger than the depth of the ASL, suggesting that the bundles only partly dip into this liquid layer (Fig. 4a). The ASL is moved cephalically by the cilia, a movement that likely also move the mucus bundles.

These bundles can be stained by Alcian blue and when observed by video microscopy they move over the surface uphill on a tilted trachea (Fig. 4b) 40, 43. There are five odd observations on these bundles and how they move that require an explanation. First, they are not moving evenly, instead they move intermittently as if they were held back by something that randomly ruptures. Secondly, they are moving slower that the ASL, on average almost 10‐times slower (Fig. 4a). Thirdly, they are held down onto the surface and are obviously not falling out into trachea on humans. Fourthly, the bundles are much thicker than the depth of the ASL and must be heavy. Lastly, they are moving perpendicular to the flow (Fig. 4b). This is unexpected for free bundles as such should be expected to move along the direction of the flow. These observations suggest that the bundles are not moving freely, but rather held back onto the tracheobronchial surface and their movement controlled. This is also logic as these should not fall off into the tracheal lumen, but rather sweep and clean the surface. How could this work?

During the passage of the last part of the submucosal gland and also when out on the tracheobronchial surface, the MUC5B mucin bundles become coated with the MUC5AC mucin secreted from the surface goblet cells 40. The MUCAC mucin seems to have a more net‐like polymer structure. This mucin can be observed reaching from inside the goblet cell up and over the MUC5B bundle (Fig. 4a). Based on these observations, we have suggested that the MUC5AC mucin anchors the MUC5B bundles to the epithelial surface.

The most severe problems with the CF disease comes from the lungs where there has been discussion on how soon after birth the lungs are affected 18. When the bundle movement was studied in newborn piglets lacking the CTFR channel, the CF pig, the bundles were almost stagnant 43. This was also observed when bicarbonate was removed. This is reminding of the observation of the CF small intestine discussed above where the mucin was not detached from its anchor to the goblet cells. When the number of goblet‐to‐bundle MUC5AC attachments was counted, these were more frequent in the CF piglet. This could suggest that the MUC5AC detachment was slower or impaired in the absence of CFTR channel activity or likely insufficient amounts of bicarbonate.

Surprisingly, we could recently observe that the addition of acetylcholine or the longer acting drug carbachol immediately stopped the bundle movement 43. As could be expected, the acetylcholine receptor antagonist Atrovent^R^ (ipratropium) reversed this effect. This compound is in clinical use for patients with chronic bronchitis and chronic obstructive pulmonary disease (COPD). The effect of this drug has been suggested to be via inhibition of bronchoconstriction caused by acetylcholine, but the observations of its effect on the bundle movement are an additional explanation for its beneficial effect.

The observations that the mucus bundles are immobile in the CF piglet further suggest that CFTR has a role in controlling mucus bundle movement, a mechanism that needs further studies. However, the observation that the CF mucus bundles do not move in newborn CF piglet has important implications as it further support the more recent understanding that the CF disease starts already at birth and that treatment, especially with correctors of CFTR should start as early as possible 18, 44.

Our observations of the mucus bundles movement and their control by acetylcholine suggest coordination of several lung functions. Acetylcholine is known to be released upon dust inhalation something that cause bronchoconstriction and an increased cilia beating frequency 38. This will increase the transport of the ASL and thus of small particles like dust. The moving with the ASL will allow dust to collect onto the stopped mucus bundles that in this way could be more efficiently loaded. The acetylcholine effect on the mucus bundles is estimated to be relatively short‐lived allowing the dust to be efficiently transported to the larynx 43. The normal cleaning of the tracheobronchial tree by mucus bundles sweeping the surface can be estimated to be very efficient, much more efficient than the till today assumed cleaning mechanism generated by mucus sheets.

## Protection of the respiratory tract by mucus in COPD and cystic fibrosis

The normal cleaning of the respiratory tract is accomplished by the efficient sweeping of the surface by the mucus bundles formed by the glandular MUC5B mucin. However, this is not what is observed in a diseased lung.

When bronchiolar lavage fluids from COPD patients were studied by proteomics methods, a number of proteins appeared that is typical for colon mucus 45. Furthermore, similar proteins are induced in a mouse model of chronic lung disease induced by elastase. Elastase is produced by leucocytes infiltrating diseased lungs and is strongly associated with severe CF disease and the development of bronchiectasis 46. Only two inhalations of elastase in mice induced goblet cell hyperplasia and the accumulation of mucus causing clogged distal airways. The proteins found in both colon and disease lungs are FCGBP, CLCA1, TGM2, DBMT1 and the mucins MUC5AC and MUC5B 45.

**Figure 5:**
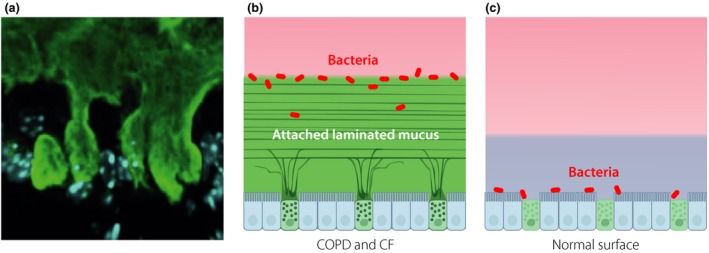
In chronic lung diseases like chronic obstructive pulmonary disease (COPD) and cystic fibrosis (CF), a mucus layer is attached to the surface epithelium. (a) Stained mucus anchored to surface goblet cells. (b) Schematic picture showing an attached laminated mucus layer where bacteria are trapped on and in the mucus. (c) On normal lungs surface, bacteria can come in direct contact with the epithelial cells. Blue layer is air surface liquid.

The mucus found in colon has a stratified appearance, also observed in the COPD and CF lungs as well as in older CF pigs and the elastase‐induced mouse model (Fig. 5) 45. Furthermore, the colon mucus is characterized by its attachment to the goblet cells, something also observed in the elastase‐induced model. This attached mucus cannot be removed by washing with PBS or hypertonic saline as used in the treatment of CF. Hypertonic saline removes most of the mucus plugging of the distal airways, but still leaves a thin mucus coat on the epithelium 45. The MUC2 mucin forms flat net‐like polymers that has the inherited tendency to form a stratified mucus 7. It is the MUC5AC mucin that most highly upregulated in both CF and COPD and as this mucin is forming net‐like polymers, more complex than MUC2, the MUC5AC mucin is likely able to form mucus layers. This means that both the MUC2 and MUC5AC mucin form structures with an inherited tendency to form stratified structures as observed in colon and diseased lungs.

Further studies of animals lacking either the MUC5B or MUC5AC mucin show that both these mouse strain can form an accumulated mucus layer and that this is dependent on both mucins 45. However, it seems as if the MUC5B mucin is more important at least in the mouse. Why this is the case is not understood, but as the MUC5B mucin from long linear polymers it could be that this is important for holding the mucus together at least in the mouse.

Why do we generate an attached and stratified mucus layer in the lungs? As this layer resembles what is found in colon where it physically separate the lumenal bacteria from the epithelial cells, this could give an explanation. In colon, the mucus layer is very important for protecting the epithelium and limit host cell contact with bacteria and by this limit tissue inflammation. Could the lung mucus have a similar function? When bacteria were instilled into normal mouse lungs, the bacteria were found to be in direct contact with the epithelial cells (Fig. 5) 45. Interestingly, in the elastase‐induced lungs with an attached surface mucus layer this trapped the bacteria in and especially on the mucus surface. This suggests that the induced mucus acted as a barrier able to separate bacteria from the epithelial cells and protect the cells also in the lung, similarly to what has been observed in colon.

Having an inducible mucus layer coating protecting the epithelial cells in the lungs is likely important and makes physiological sense as a way to protect the tissue (Fig. 5). However, this requires that the protection is only temporary and that the mucus coating is removed once the infection is under control. Removing the accumulated mucus layer require that it is released from its attachment to the epithelium. The mucus also needs to be transported, but as the mucus sheets might be both thick and large the normal ciliary beating might be insufficient. Instead, large mucus assemblies will require coughing to be transported. This is in fact what is happening for most individuals as the final outcome of bronchitis infection mucus is coughing.

If the mucus is not possible to mobilize and remove by coughing, as in CF and COPD, the attached mucus will remain attached to the epithelium. Bacteria trapped in the mucus will remain in the lungs. Certain bacteria, like *Pseudomonas aeruginosa*, seem to have a specific tendency to colonize such mucus in especially CF 47. Bacteria colonizing the attached mucus and remaining in the lungs will trigger the host immune system and the generated inflammation will contribute to the destruction of the lungs as is observed in both CF and COPD. As long as the mucus remains anchored to the epithelial surface, this will be limited options to clear the lungs from bacteria.

We now need to understand the molecular mechanisms for the formation of the lung mucus layer and its attachment in diseased lungs. Most important is to understand how detachment of the mucus can take place in the normal individual and how this is controlled. CFTR and its activity is likely an important part of this. From what we know today, turning off CFTR will generate an attached mucus layer and that its activation help to detach the mucus. The CFTR mediated bicarbonate secretion might be important for this detachment and its chloride secreting capacity to generate fluid that flush the tracheobronchial surface.

## Conclusions

Although mucus has often been considered passive and boring, recent understanding has proven mucus to be highly dynamic and to explain both normal physiology and several diseases. The complexity of the mucins building the structural skeleton of mucus explains the slow development of our understanding. However, we can now foresee a faster development in the molecular understanding of mucus paving the road for a number of novel therapies for both common and rare diseases of the lung and intestine. Examples of potential options for treating mucus related diseases are given in Table 2.

**Table 2:** Potential options for treating mucus‐related diseases

Organs	Desired effect	Potential treatments
Colon	Improved mucus barrier	Improved mucus properties by small molecule
Colon	Improved mucus barrier	Specific bacteria, prebiotics
Colon	Improved mucus barrier	Ion channel drug
Small intestine	Detach CF mucus	Ion channel drug, calcium chelator
Lung‐nose	Detach mucus bundles	Anticholinergic drug^a^
Lung‐nose	Detach mucus bundles	Ion channel drug, calcium chelator
Lung‐nose	Detach thick mucus layer	Calcium chelator, mucin interaction inhibitor, ion channel drug
Mouth	Mucus replacement	Synthetic mucins/mucus

CF, cystic fibrosis.

a In use: ipratropium.

## Conflict of interest statement

No conflict of interest was declared.
